# Pro-inflammatory and counter-regulatory modulators of interleukin-5–driven eosinophil programs: a framework for precision medicine in eosinophilic diseases

**DOI:** 10.3389/fimmu.2026.1836661

**Published:** 2026-04-14

**Authors:** Hisashi Sasaki, Jun Miyata, Koichi Fukunaga

**Affiliations:** 1 Division of Infectious Diseases and Respiratory Medicine, Department of Internal Medicine, National Defense Medical College, Saitama, Japan; 2 Division of Pulmonary Medicine, Department of Medicine, Keio University School of Medicine, Tokyo, Japan

**Keywords:** eosinophil, IL-13, IL-33, IL-4, IL-5, interferon, NOD2, retinoic acid

## Abstract

Interleukin-5 (IL-5) is central to eosinophil differentiation, survival, and activation. Subsequent studies confirmed IL-5 receptor expression and mapped downstream signaling, showing that IL-5 promotes not only survival but also trafficking and effector functions, including adhesion, degranulation, reactive oxygen species generation, mediator release (cytokines and cysteinyl leukotrienes), and extracellular trap formation. However, the roles of eosinophils vary across diseases and tissue compartments. Multi-omics analyses of blood and tissue eosinophils across transcriptomic, proteomic, and lipid-metabolic layers reveal disease- and site-specific molecular states. These data support two opposing classes of modulators that shape IL-5–driven programs: pro-inflammatory signals (IL-4/IL-13, IL-33, NOD2-mediated innate signaling, and IFN-γ) that drive inflammatory eosinophil changes and can cooperate in some settings, and counter-regulatory signals (IFN-α and all-trans retinoic acid [ATRA]) that restrain these changes. Such modulation may influence tissue retention, effector functions, and broader eosinophil activities, including antiviral and homeostatic roles. Clinical studies of anti-IL-5 and/or anti-IL-5Rα biologics in severe eosinophilic asthma, chronic rhinosinusitis with nasal polyps, eosinophilic granulomatosis with polyangiitis, hypereosinophilic syndromes, and other eosinophilic diseases have improved outcomes, underscoring that disease activity often depends on IL-5-driven eosinophil activation despite disease-specific IL-5–independent signals. In this review, we summarize IL-5 biology from mechanisms to therapy and discuss how integrated multi-omics signatures and clinical biomarkers may guide patient stratification, therapy selection, and treatment sequencing toward precision medicine for eosinophilic respiratory and systemic diseases.

## 1 Introduction

Refractory eosinophilic diseases are often characterized by prominent type 2 inflammation. This inflammatory phenotype is driven by coordinated actions of type 2 cytokines produced by lymphocytes such as Th2 cells and group 2 innate lymphoid cells (ILC2s), including IL-5, IL-4, and IL-13, and epithelial-derived cytokines such as IL-33, IL-25, and thymic stromal lymphopoietin (TSLP). Within this network, IL-5 plays a central role in promoting eosinophil accumulation and activation at inflamed sites, thereby contributing to tissue injury (1). Activated tissue eosinophils can induce organ damage through degranulation, reactive oxygen species production, cytokine release, cysteinyl leukotriene (Cys-LT) synthesis, and extracellular trap formation (2). In the airways, these processes are linked to bronchoconstriction, increased airway hyperresponsiveness, airway remodeling, mucus plugging, and nasal polyp formation. IL-5 receptor expression is most prominent on eosinophils, but expression has also been reported on basophils and other immune cell subsets (e.g., plasma cells and mast cells), and in some studies on structural cells such as epithelial cells and fibroblasts, suggesting additional pathways by which IL-5 may amplify inflammation. Together, these observations support the view that IL-5 is a key organizer of eosinophil-centered immune responses in type 2 inflammation (3).

Therapeutic targeting of the IL-5 pathway has demonstrated clinical benefit in several eosinophilic disorders. Anti-IL-5 and anti-IL-5Rα biologics, including mepolizumab, reslizumab, benralizumab, and more recently depemokimab, have improved outcomes across several eosinophilic disorders, particularly severe eosinophilic asthma, CRSwNP, EGPA, HES, and selected other eosinophilic lung diseases (4, 5). These therapeutic effects, observed across clinically diverse conditions, support a central role for IL-5 and eosinophils in disease pathogenesis. In practice, blood eosinophil counts and sputum eosinophilia are commonly used as biomarkers of eosinophilic inflammation and are associated with responsiveness to IL-5 pathway biologics (6). However, eosinophilia does not consistently predict treatment benefit, and robust approaches to capture IL-5-dependent pathogenic activation of eosinophils in individual patients remain insufficiently established. Also, this gap is particularly important because eosinophils with similar abundance may differ substantially in their activation state, tissue adaptation, and effector programs depending on the surrounding cytokine and inflammatory milieu. An integrated multi-omics framework is therefore needed to define these context-dependent IL-5-conditioned eosinophil states more precisely and to advance patient stratification, therapy selection, and treatment sequencing beyond simple eosinophil counts.

Eosinophils respond strongly to IL-5, yet they also express a broad range of receptors and integrate diverse signals (7). In addition to IL-5, the related cytokines GM-CSF and IL-3 share the common β chain and can induce overlapping downstream programs, while eotaxins trigger strong chemotaxis through CCR3 (3, 8). Cytokines often discussed in the context of lymphocyte activation, such as IL-4/IL-13 and IL-33, can also directly modulate eosinophil activation (9, 10). Signals associated with type 1 and type 3 inflammation, including interferons, and innate sensing pathways triggered by microbial molecules (including formyl peptides such as N-formylmethionyl-leucyl-phenylalanine (fMLP) and pattern-recognition receptor pathways such as Toll-like receptors (TLRs) and nucleotide-binding oligomerization domain–like receptors (NOD-like receptors; NLRs) have also been implicated in shaping eosinophil functions (9, 11, 12). Lipid mediators such as prostaglandin D_2_ (PGD_2_), prostaglandin E_2_ (PGE_2_), 5-oxo-ETE, and platelet-activating factor (PAF) are well-established regulators of eosinophils (13). In addition, fat-soluble metabolites, including vitamin A metabolites such as all-trans retinoic acid (ATRA), have been reported to influence eosinophil programs (14). Therefore, how these signals modify IL-5-conditioned eosinophils may be a key determinant of inflammatory direction, tissue pathology, and variable responses to therapy.

This review summarizes the history and biology of the IL-5/IL-5R axis and core IL-5-dependent eosinophil functions, then discusses how representative cytokine, innate-sensing, interferon, and lipid/vitamin-derived signals modulate eosinophil phenotypes. Mechanistic studies, together with multi-omics profiling, can reveal how specific signaling inputs reshape IL-5-conditioned eosinophil programs and thereby help explain clinical heterogeneity. Finally, the review considers how these concepts may inform biomarker-guided patient stratification and rational selection and sequencing of therapies for eosinophilic respiratory and systemic diseases.

## 2 Biology of IL-5 and its receptor: history, structure, and signaling

### 2.1 Discovery and molecular features of IL-5

IL-5 was first identified in mouse studies as a T cell-derived lymphokine with two reported activities: a B cell growth and differentiation factor and an eosinophil colony-stimulating factor (15). Subsequent biochemical purification and molecular cloning showed that both activities are mediated by a single cytokine, now designated IL-5, which forms a disulfide-linked homodimer and belongs to the four-helix bundle family (16). IL-5 is relatively conserved between mice and humans, supporting the use of experimental models to study human disease.

The human *IL5* gene is located on chromosome 5q31–33 within a type 2 cytokine gene cluster that also includes *IL4* and *IL13*, consistent with coordinated regulation during type 2 immune responses (17, 18). During Th2 differentiation, *IL5* expression is regulated by type 2 transcription factors, including GATA3 and STAT5, which integrate antigen stimulation, cytokine signals, and environmental cues to drive type 2 cytokine production (19, 20). IL-5 is produced mainly by Th2 cells and ILC2s, and mast cells have also been reported as an additional source in certain inflammatory settings, supporting eosinophilopoiesis, survival, and priming *in vivo* (3, 8).

### 2.2 Structure and signaling of the IL-5Rα/βc receptor complex

IL-5 signals through a heteromeric receptor composed of a cytokine-specific α chain (IL-5Rα, encoded by *IL5RA*) and the common β chain (βc), which is shared with the receptors for GM-CSF and IL-3 (21). IL-5Rα provides ligand specificity and high-affinity binding, whereas βc is required for signal transduction and helps stabilize the active receptor complex (21). Structural studies have clarified how IL-5 is recognized and how receptor activation occurs. X-ray crystallography of human IL-5 bound to the IL-5Rα ectodomain showed the key binding interfaces between IL-5 and IL-5Rα, which are relevant to neutralizing antibodies and receptor variants (22). More recently, cryo–electron microscopy of the ternary complex (IL-5, IL-5Rα, and βc) revealed how IL-5-IL-5Rα binding enables βc recruitment and productive receptor assembly, consistent with a shared activation mechanism across the βc cytokine receptor family (23).

On the cytoplasmic side, receptor assembly activates Janus kinases and downstream signaling pathways. βc-associated kinases promote tyrosine phosphorylation and recruitment of STAT proteins (notably STAT5, with context-dependent activation of STAT1 and STAT3), as well as signaling through PI3K/AKT and MAPK pathways (24–26). Through these pathways, IL-5 supports eosinophil survival, increases responsiveness to chemokines and lipid mediators, and regulates adhesion and trafficking.

IL-5 signaling is limited by negative-feedback mechanisms, including induction of SOCS family proteins and regulation of receptor turnover. For example, phosphorylation-dependent ubiquitination and internalization of the receptor complex can shorten signaling, and JAK activity has been implicated in controlling IL-5R ubiquitination and degradation (27). Crosstalk with other βc cytokines (GM-CSF and IL-3) can further shape signaling output; these cytokines share core pathways with IL-5, including JAK/STAT, PI3K/AKT, and MAPK signaling, but their upstream signaling requirements and downstream functional effects are not completely identical (28, 29). In particular, IL-3 may show partial divergence from IL-5 and GM-CSF in some systems, supporting the view that βc cytokines are broadly overlapping but not fully interchangeable in signaling behavior (29).

## 3 Canonical roles of IL-5 in eosinophil biology

This section summarizes the canonical effects of IL-5 on eosinophils. These include enhanced eosinophilopoiesis, prolonged survival, increased trafficking to inflamed tissues, and priming of effector functions that can contribute to airway injury and remodeling.

### 3.1 Eosinophilopoiesis: eosinophil differentiation and maturation in bone marrow

IL-5 is a central determinant of eosinophil lineage expansion and terminal maturation. Genetic and transgenic mouse studies established that IL-5 is largely dispensable for global myelopoiesis yet non-redundant for expanding eosinophil lineage–committed progenitors and driving peripheral eosinophilia (30). In humans, IL-5 supports eosinophil production from CD34^+^/IL-5Rα^+^ marrow progenitors, acting alongside IL-3 and GM-CSF to promote maturation and yield (31).

Mechanistically, IL-5 can function as an initiating cue that establishes a pro-eosinophilic cytokine milieu within the marrow niche (32). In low-density bone marrow culture systems, 4 days of IL-5 exposure were sufficient to trigger a cooperative cytokine network that continued to promote eosinophil precursor maturation even after IL-5 withdrawal, with ongoing expansion and differentiation observed for at least an additional 48 hours in the absence of further IL-5 stimulation (33). This included induction of accessory pathways (e.g., IL-4/IL-4Rα and chemokine loops such as CCL3–CCR1) capable of sustaining terminal differentiation in the absence of continuous IL-5. Together, these observations refine the classical view of IL-5 as a simple eosinophilopoietic factor, instead positioning it as a trigger that installs a durable eosinophil-permissive program. Although residual eosinophils during IL-5-targeted therapy are partially consistent with this concept, a comparable memory-like effect has not been directly established *in vivo*. Nevertheless, this framework suggests that eosinophil development and persistence may be supported, at least in part, by IL-5-independent signals, including related βc cytokines.

### 3.2 Survival, trafficking, and tissue retention: Bcl-2 family, chemokines, and adhesion molecules

Beyond differentiation, IL-5 prolongs eosinophil lifespan by suppressing intrinsic apoptosis and maintaining pro-survival signaling. A key mechanism is induction of the anti-apoptotic Bcl-2 family member Bcl-xL: IL-5 (and related βc cytokines) delays constitutive apoptosis and apoptosis after cytokine withdrawal through upregulation of Bcl-xL (34). *In vivo* genetic studies further link IL-5-mediated survival to NF-κB-dependent regulation of Bcl-xL, supporting an IL-5-NF-κB-Bcl-xL axis as an important determinant of eosinophil longevity (34).

IL-5 also promotes eosinophil trafficking by increasing chemokine responsiveness and integrin-mediated adhesion. In the circulation, IL-5 enhances CCR3-dependent responses to eotaxins and related CC chemokines, while in inflamed tissues it cooperates with endothelial adhesion molecules (VCAM-1 and ICAM-1) and eosinophil integrins (e.g., α4β1 and β2 integrins) to support firm adhesion and transendothelial migration into type 2–inflamed tissues (35–37). Once in tissues, continued exposure to locally produced IL-5 and other survival factors, together with ongoing integrin-mediated adhesion to structural elements of the tissue microenvironment, promotes eosinophil retention and persistence (38, 39). In this setting, the local tissue microenvironment may sustain pro-survival programs, including NF-κB/Bcl-xL-associated signaling, even during systemic IL-5 blockade. Thus, this model is consistent with the clinical observation that IL-5 blockade rapidly depletes circulating eosinophils but may incompletely eliminate tissue-resident eosinophils in severe airway and systemic eosinophilic diseases (40–42), suggesting that local survival niches contribute to persistent tissue eosinophilia.

### 3.3 Effector functions: degranulation, lipid mediator synthesis, and ETosis/EETs

IL-5 primes eosinophils for stronger effector responses by lowering the activation threshold and increasing responses to secondary stimuli, such as Fc receptor engagement, chemokines (including eotaxins), complement factors (e.g., C5a), and lipid mediators (e.g., PAF) (43, 44). In this IL-5-conditioned state, eosinophils more readily release granule proteins and produce lipid mediators, including Cys-LTs, which can contribute to bronchoconstriction, mucus hypersecretion, and increased vascular permeability (43).

A major advance has been the recognition of eosinophil extracellular trap cell death (ETosis) and eosinophil extracellular traps (EETs) as disease-relevant effector programs that can be facilitated by IL-5 priming. Activated human eosinophils can undergo ETosis and release extracellular chromatin structures decorated with granule proteins, in an NADPH oxidase–dependent manner (45). EETs have been observed in eosinophil-rich secretions, such as nasal mucus in CRSwNP and sputum in severe asthma, where they retain cytotoxic granule proteins and may promote persistent inflammation, nasal polyp formation, and mucus plugging (46). Importantly, IL-5 alone is usually insufficient to induce ETosis; instead, IL-5 priming can enhance ETosis in response to secondary stimuli, including PAF or CCL11 (45).

### 3.4 IL-5–driven eosinophilia causes airway dysfunction and remodeling: evidence from mouse models

Mouse models provide strong evidence that IL-5-driven eosinophilia can cause airway dysfunction. Lung-restricted overexpression of IL-5 induces spontaneous peribronchial eosinophilic inflammation and is associated with airway remodeling, including goblet cell metaplasia, epithelial changes, collagen deposition, and airway hyperresponsiveness (47). Conversely, in chronic allergen models, IL-5 deficiency markedly reduces airway eosinophilia and attenuates remodeling features such as peribronchial fibrosis, smooth muscle hypertrophy, and TGF-β activation (48). Adoptive transfer studies further support a causal role for eosinophils, as transferred eosinophils can restore aspects of airway hyperresponsiveness and remodeling in IL-5-deficient settings. Taken together, these models link IL-5-dependent eosinophilia to airway dysfunction and structural remodeling and support eosinophils as key downstream effectors.

## 4 Signals beyond IL-5 that shape eosinophil phenotypes

Disease-specific eosinophil phenotypes emerge when additional signals modify the IL-5-conditioned baseline and shift which effector functions become dominant. In this chapter, we summarize the direct effects of these signals on eosinophils, with multi-omics readouts briefly illustrating molecular eosinophil profiles.

### 4.1 Sources of context signals: alarmins, microbes, and retinoids in type 2 inflammation

Epithelial-derived alarmins, including IL-33, IL-25, and TSLP, promote type 2 inflammation by expanding and activating ILC2s and Th2 cells, thereby driving production of type 2 cytokines such as IL-5 and IL-13/IL-4. In many allergic diseases where type 2 inflammation is central to pathology, exposure to or colonization by microbes—including bacteria, fungi, and viruses—can exacerbate disease, in part because microbial components directly stimulate immune cells and amplify local inflammation (49). Importantly, microbial exposure can also increase epithelial release of IL-33, in some cases through microbial protease activity, further strengthening type 2 cytokine circuits (50). In contrast, retinoids act as homeostatic regulators of mucosal immunity: vitamin A-derived metabolites are continuously generated in tissues by resident macrophages and dendritic cells and help restrain excessive immune responses (51). Together, these observations highlight that eosinophils in inflamed tissues are exposed not only to IL-5 but also to a broader network of context signals. Therefore, identifying the drivers of eosinophil pathogenicity requires understanding how combined inputs from alarmins, microbial cues, and regulatory metabolites reshape eosinophil states and functions *in vivo*.

### 4.2 IL-4/IL-13

IL-4 and IL-13 are central type 2 cytokines that promote epithelial mucus programs and tissue remodeling and, particularly for IL-4, support IgE-skewed humoral immunity. In addition to these well-established tissue-level effects, prior *in vitro* studies indicate that IL-13 can act directly on human eosinophils, inducing activation markers such as CD69 and supporting viability, as well as prolonging survival and promoting chemotaxis (52, 53).

Building on these observations, our multi-omics profiling suggests that IL-4 (and similarly IL-13) does not simply increase eosinophil activation in a uniform manner but rather reprograms IL-5-primed eosinophils into a distinct state. This program is characterized by increased expression of cytokine receptor genes (including IL1RL1 and IL3RA) and enhanced lipid mediator–related enzymes (including transglutaminase 2 [TGM2] and gamma-glutamyltransferase 5 [GGT5]), which were among the most prominently induced markers in IL-4/IL-13-conditioned eosinophils, consistent with an additive increase in cysteinyl leukotriene synthesis, most notably LTD_4_, compared with IL-5 alone (9). Importantly, similar molecular features were also observed in eosinophils isolated from nasal polyps of patients with CRSwNP, supporting the disease relevance of this IL-4/IL-13–associated signature (9). This lipid-metabolic shift may contribute to airway inflammatory responses, providing a potential mechanistic link to the nasal polyp or mucus plugging phenotype observed in CRSwNP or asthma.

Together, these findings support a complementary model in which IL-5 sustains eosinophil abundance and baseline readiness, whereas IL-4/IL-13 reshape effector programs by linking eosinophils to epithelial alarmin circuits and amplifying lipid mediator pathways.

### 4.3 IL-33

IL-33 is an epithelial-derived alarmin that strengthens type 2 inflammation upstream by activating ILC2s and increasing IL-5 production, and it also acts directly on mature eosinophils. Through its receptor IL1RL1, IL-33 activates human eosinophils and promotes superoxide production, degranulation, and survival, while increasing adhesion capacity and further prolonging eosinophil survival (54, 55). Mechanistic studies have also linked IL-33 signaling to eosinophil lineage commitment, tissue activation, and progenitor dynamics (56).

Our data suggest that eosinophil responses to IL-33 are shaped by the local cytokine milieu. IL-4/IL-13 conditioning enhances IL1RL1-associated programs and strengthens IL-33-mediated survival, indicating that type 2 cytokines tune both the magnitude and downstream consequences of IL-33 signaling in tissues (9). In our multi-omics analyses, IL-33 induced a molecular expression profile distinct from IL-5, including increased expression of IL1A/IL1B, CCL3/CCL4, ICAM1, CD4, and CD22, and IL-5 co-stimulation induced an inflammatory lipid-metabolic phenotype, marked by increased GGT5 and reduced DPEP2 (10). Importantly, similar changes were observed in eosinophils isolated from nasal polyps of patients with CRSwNP, supporting the disease relevance of this IL-33-associated state (10).

In addition, IL-33 alone triggered eosinophil extracellular trap formation (ETosis) in our system. Because IL-5 by itself is a weak inducer of ETosis, these findings suggest that epithelial alarmins such as IL-33 provide critical inputs that, together with IL-5, promote pathogenic eosinophil programs in inflamed tissues.

### 4.4 NOD2

Innate sensing pathways represent a distinct mechanism by which eosinophil states can be remodeled in inflamed tissues. Human eosinophils express innate receptor machinery, including TLRs (e.g., TLR2, TLR4, TLR5, and TLR7/8) and NLRs (e.g., NOD1 and NOD2). NODs are intracellular pattern-recognition receptors that detect microbial ligands and activate inflammatory signaling (57, 58). NOD1/2 ligation modulates eosinophil activation *in vitro* by inducing IL-8 release, altering adhesion/activation marker expression, promoting migration, and triggering eosinophil-derived neurotoxin release (59).

Extending these findings into a disease-relevant fungal context, our multi-omics study showed that *Aspergillus fumigatus* extract induces a NOD2-dependent and oxidative stress–associated activation module in eosinophils, including coordinated changes in CD62L, CD11b, and CD69. NOD2-driven gene signatures were distinct from those induced by IL-5 alone, yet exhibited synergistic changes with IL-5, consistent with potentiation of IL-5-conditioned activation programs (12). Notably, NOD2 transcript levels were increased in blood eosinophils from patients with severe asthma compared with those from healthy participants (14).

Collectively, these findings suggest that in clinical settings where microbial-triggered innate responses overlap with type 2 eosinophilic inflammation, NOD2- and oxidative stress–linked activation can cooperate with IL-5 priming to amplify eosinophil pathogenic programs in tissues. In this context, increased eosinophil NOD2 expression may indicate susceptibility to microbe-associated exacerbation pathways. However, because NOD2 is an intracellular receptor, its direct clinical use as a biomarker may be limited. Future studies are needed to identify accessible surrogate biomarkers of NOD2-associated eosinophil activation for patient stratification.

### 4.5 IFN-γ

IFN-γ is a key cytokine in antimicrobial, type 1 immunity and is often viewed as counter-regulatory to type 2 inflammation at the systemic level (60). However, eosinophils can respond directly to IFN-γ in ways that promote inflammation, particularly in chronic disease or infection-associated contexts. Specifically, IFN-γ has been reported to prolong eosinophil survival, increase cysteinyl leukotriene production, enhance the expression or release of inflammatory mediators (including IL-3, MIG/CXCL9, and IP-10/CXCL10), promote RANTES (CCL5) release, and increase eosinophil cationic protein (ECP) expression (61–63).

In our multi-omics profiling, IFN-γ induced antiviral programs in eosinophils, including upregulation of guanylate-binding proteins (GBPs) and tripartite motif-containing proteins (TRIMs), which are interferon-inducible antiviral effector families, and increased expression of adhesion-related molecules (including ICAM1) and Fc receptor components (including FCGR1A) (11). Notably, IFN-γ restored IL5RA expression that is downregulated by chronic IL-5 exposure, suggesting a mechanism by which IFN-γ may help sustain IL-5 responsiveness within inflamed tissues (11). This observation aligns with prior evidence that IFN-γ can enhance IL-5–mediated eosinophil responses (64).

In airway inflammation, type 2 pathways can coexist with intermittent infection-related signals. In this context, IFN-γ can remodel IL-5-conditioned eosinophil states and shape their effector programs, rather than simply opposing type 2 inflammation.

### 4.6 IFN-α

Type I interferons, including IFN-α, orchestrate broad antiviral programs and can counterbalance inflammatory persistence (65). Consistent with this role, earlier work reported that IFN-α can suppress eosinophil degranulation, indicating a direct inhibitory effect on eosinophil effector function (66). In our study, IFN-α induced a robust antiviral transcriptional program in eosinophils, accompanied by increased expression of TRIMs and GBPs, and it antagonized IL-5–mediated long-term survival. These findings are consistent with a constraint layer in which interferon-rich environments may limit the longevity and effector persistence of IL-5-conditioned eosinophils (11).

This contrast may be particularly relevant during virus-associated exacerbations in asthma. Type I interferons primarily support antiviral defense and may restrain eosinophil effector persistence, whereas IFN-γ can promote inflammatory remodeling of IL-5-conditioned eosinophils and potentially amplify type 2–skewed responses in infection-associated settings. Together, these distinct interferon effects may help explain why viral infection can both trigger antiviral immunity and, in susceptible patients, precipitate acute worsening of type 2 airway inflammation.

### 4.7 ATRA

Retinoids regulate mucosal immunity through nuclear receptor signaling and can exert context-dependent effects on eosinophil biology. ATRA contributes to immune tolerance, including induction of regulatory T cells and regulatory ILCs (67). In eosinophils, prior mechanistic studies have shown that ATRA can increase expression of the chemokine receptor CCR3 (68) and inhibit spontaneous apoptosis (69), indicating potential effects on trafficking competence and survival. At the same time, retinoid signaling is increasingly recognized to be context dependent, with both pro- and anti-inflammatory consequences across immune settings (70).

Our multi-omics analyses provide disease-state refinement of these concepts. Compared with blood eosinophils from healthy donors, blood eosinophils from patients with severe asthma showed reduced expression of ATRA-associated factors, including SPRY2 and HIC1. In the same cells, inflammatory programs were increased, with higher IL-5–induced gene expression of GGT5, IL2RA, and CCL23, and increased protein expression of PSGL-1. Notably, ATRA suppressed IL-5–driven upregulation of GGT5 and PSGL-1, indicating direct antagonism of IL-5-conditioned activation (14). At the lipid-metabolic level, ATRA reduced Cys-LT production, while having minimal effect on 15-lipoxygenase–linked pathways implicated in pro-resolving lipid mediator generation. In addition, eosinophils in the long-term presence of ATRA displayed markedly reduced expression of IL1RL1, IL3RA, and NOD2, consistent with attenuated responsiveness to IL-33 and IL-3 (14).

Together, these findings support retinoic acid signaling as a counter-regulatory layer that restrains IL-5–driven inflammatory programs and shifts eosinophils toward a less alarmin- and innate cue–responsive state. Under retinoid-rich conditions, eosinophils may therefore favor homeostatic/regulatory functions.

### 4.8 IL-5–centered integrative model

Collectively, the evidence in this section supports a simple model in which IL-5 establishes a baseline eosinophil program (expansion, survival, tissue recruitment, and priming) while additional signals determine how eosinophils behave in each tissue and disease. Signals that commonly amplify or redirect IL-5-primed eosinophils include type 2 cytokines and alarmins (IL-4/IL-13 and IL-33), microbial/innate sensing pathways (including NOD2–linked modules), and IFN-γ. These inputs can increase adhesion and trafficking capacity, enhance inflammatory mediator production, and promote injury-associated programs such as extracellular trap formation, thereby strengthening eosinophil-driven inflammation in affected tissues. In contrast, counter-regulatory pathways such as type I interferon and ATRA can limit eosinophil effector persistence and dampen specific IL-5-conditioned inflammatory programs.

**Figure 1:**
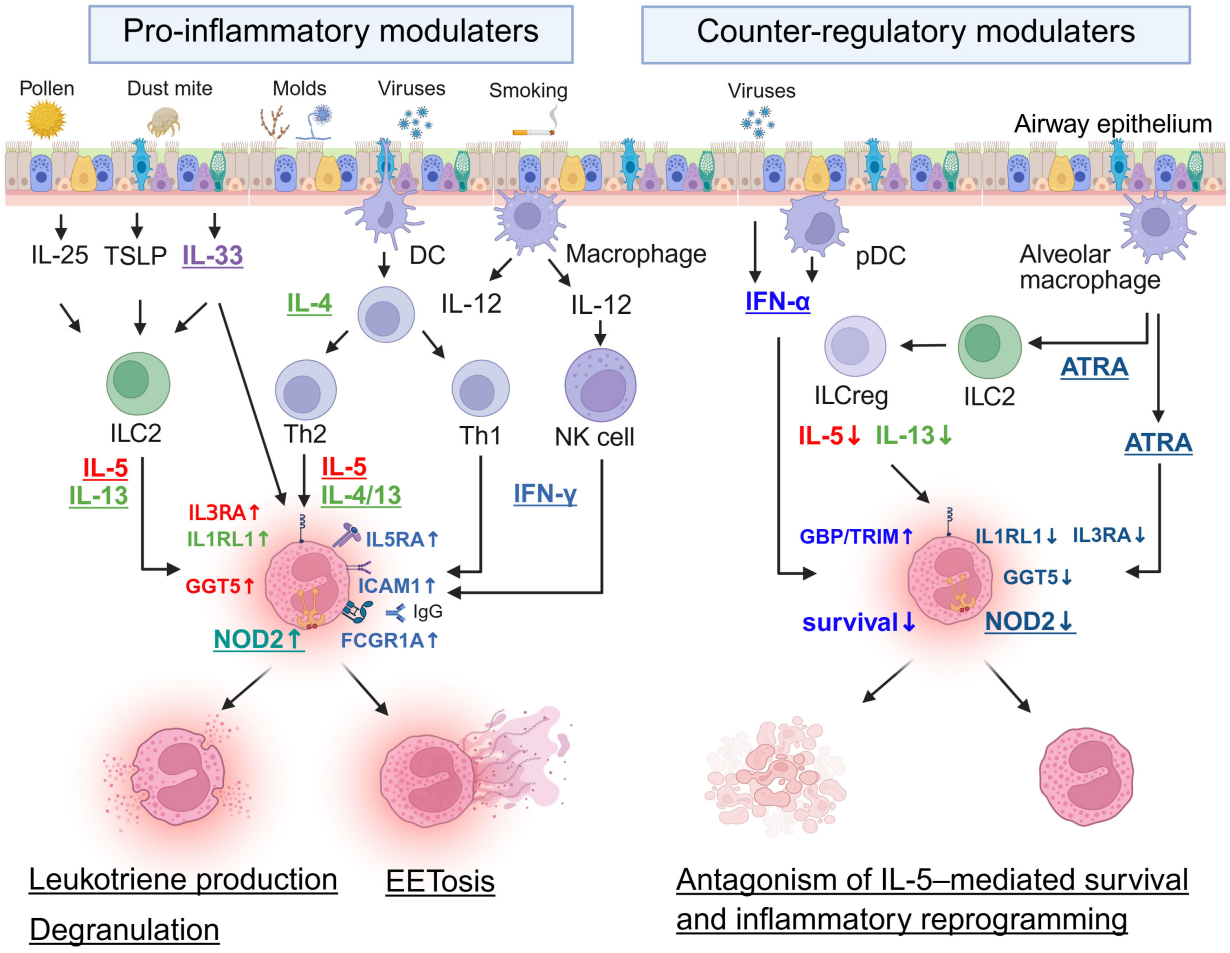
Pro-inflammatory and counter-regulatory modulators that shape IL-5-driven eosinophil responses. IL-5 provides a baseline eosinophil state (survival, priming, and readiness for tissue inflammation), while additional context-dependent signals shift eosinophil responses toward inflammatory amplification or counter-regulation. Pro-inflammatory modulators at the airway epithelial interface (e.g., allergens, microbes/viruses, and smoking-related stimuli) promote epithelial alarmins (IL-25, TSLP, IL-33) and immune pathways involving ILC2/Th2 (IL-5, IL-4/IL-13) as well as IL-12/IFN-γ-associated circuits, together with NOD2-related innate signaling. These inputs enhance inflammatory eosinophil features (e.g., IL3RA, IL1RL1, GGT5, NOD2, IL5RA, ICAM1, FCGR1A) and downstream effector functions, including leukotriene production, degranulation, and EETosis. In contrast, counter-regulatory modulators, including type I IFN (IFN-α) and ATRA, limit IL-5–driven eosinophil persistence and inflammatory activation by inducing antiviral pathways (e.g., GBP/TRIM) and suppressing selected alarmin/innate response-associated molecules (e.g., IL1RL1, IL3RA, GGT5, NOD2). ATRA may also support ILCregs and restrain type 2 effector circuits, consistent with a broader tissue counter-regulatory environment. ATRA, all-trans retinoic acid; DC, dendritic cell; EETosis, eosinophil extracellular trap cell death; FCGR1A, Fc gamma receptor 1A; GBP, guanylate-binding protein; GGT5, gamma-glutamyltransferase 5; ICAM1, intercellular adhesion molecule 1; IFN-α, interferon-alpha; IFN-γ, interferon-gamma; IL, interleukin; IL1RL1, interleukin 1 receptor-like 1; IL3RA, interleukin 3 receptor subunit alpha; IL5RA, interleukin 5 receptor subunit alpha; ILC2, group 2 innate lymphoid cell; ILCregs, regulatory innate lymphoid cells; NK, natural killer; NOD2, nucleotide-binding oligomerization domain-containing protein 2; pDC, plasmacytoid dendritic cell; Th1, type 1 helper T cell; Th2, type 2 helper T cell; TRIM, tripartite motif-containing protein; TSLP, thymic stromal lymphopoietin.

This concept provides a mechanistic explanation for why blocking the IL-5 pathway can consistently reduce eosinophilic inflammation and improve disease activity across diverse eosinophilic disorders, even when the upstream immune environment differs among diseases. It also suggests that mixed Th1/Th2 conditions do not necessarily preclude responsiveness to IL-5 blockade, because IFN-γ may help sustain IL-5 responsiveness, whereas in infection-associated settings, concurrent management of microbial triggers may also be relevant. **Figure 1** summarizes the principal modulators discussed in this section, their major cellular sources, and their direct effects on eosinophil state programs.

## 5 IL-5–eosinophil axis in eosinophilic diseases and therapeutic targeting

Anti-IL-5 and anti-IL-5Rα therapies have shown clinical benefit across multiple eosinophilic diseases, including SEA, CRSwNP, EGPA, HES, and other eosinophilic diseases such as allergic bronchopulmonary aspergillosis/mycosis (ABPA/ABPM) and chronic eosinophilic pneumonia (CEP). Although these disorders differ in key pathogenic features, such as the balance of innate and adaptive immune pathways, the contribution of viruses, bacteria, or fungi, and the presence or absence of autoimmune inflammation, they commonly share marked tissue eosinophilia and elevated IL-5 levels at sites of disease. The efficacy of IL-5-targeted therapy across this heterogeneous spectrum suggests that IL-5 functions as a central axis that amplifies eosinophilic inflammation in concert with other disease-driving factors, including IL-4/IL-13, epithelial alarmins such as IL-33, ligands for pattern-recognition receptors (e.g., NOD2-related pathways), and IFN-γ. In this section, the clinical evidence for IL-5 pathway blockade is summarized by disease, focusing on pivotal trials and clinically relevant outcomes; key studies are also compiled in **Table 1**.

**Table 1:** IL-5-targeted biologic therapies across eosinophilic diseases.

Disease	Biologics	Target	Evidence type	Key outcomes	Ref
SEA	Mepolizumab	IL-5	Pivotal RCT	Reduced exacerbations; improved PROs; OCS–sparing	(71, 72)
	Mepolizumab	IL-5	Study (physiology/remodeling)	Improved fixed airflow limitation; Attenuated airway remodeling	(73)
	Depemokimab	IL-5	Phase 3 RCT	Clinically meaningful exacerbation reductions with twice-yearly dosing	(77)
	Benralizumab	IL-5Rα	Pivotal RCT	Reduced exacerbations; improved lung function/symptoms; OCS–sparing	(74–76)
CRSwNP	Mepolizumab	IL-5	Phase 3 RCT	Reduced endoscopic nasal polyp size; improved nasal obstruction	(80)
	Depemokimab	IL-5	Phase 3 RCT	Sustained reductions in nasal polyp score and improved nasal obstruction	(81)
EGPA	Mepolizumab	IL-5	Phase 3 RCT	Increased remission; reduced relapses; OCS reduction	(85)
	Mepolizumab	IL-5	Observational/case-based	Improved peripheral neuropathy	(87)
	Mepolizumab	IL-5	Case series/literature review	Declines in MPO-ANCA titers	(88)
	Benralizumab	IL-5Rα	Phase 3 RCT	Increased remission; reduced relapses; OCS reduction	(86)
HES	Mepolizumab	IL-5	Phase 3 RCT	Reduced disease flares; improved overall disease control	(89)
	Benralizumab	IL-5Rα	Phase 2/3 RCT	Reduced disease flares; improved overall disease control (NATRON)	(90)
ABPA/ABPM	Benralizumab	IL-5Rα	Observational cohorts/case series	Reduced mucus plugs; fewer relapses; improved lung function; OCS-sparing	(93)
CEP	IL-5-targeted biologics	IL-5/IL-5Rα	Case series	Improved disease activity; reduced relapse risk; OCS-sparing	(94)

SEA, severe eosinophilic asthma; CRSwNP, chronic rhinosinusitis with nasal polyps; EGPA, eosinophilic granulomatosis with polyangiitis; HES, hypereosinophilic syndrome; ABPA/ABPM, allergic bronchopulmonary aspergillosis/mycosis; CEP, chronic eosinophilic pneumonia; IL, interleukin; IL-5Rα, IL-5 receptor alpha; RCT, randomized controlled trial; PROs, patient-reported outcomes; OCS, oral corticosteroids; SCS, systemic corticosteroids; MPO-ANCA, myeloperoxidase anti-neutrophil cytoplasmic antibody.

### 5.1 SEA

SEA is a difficult-to-control asthma phenotype characterized by persistent type 2 inflammation and frequent exacerbations, often accompanied by elevated blood and/or airway eosinophils. In severe eosinophilic asthma, IL-5 pathway biologics provide broad clinical benefits, including robust suppression of exacerbations, improvements in lung function, better symptom control and health-related quality of life, and clinically meaningful oral corticosteroid (OCS)–sparing effects.

For mepolizumab, pivotal studies such as MENSA and MUSCA demonstrated marked reductions in exacerbations together with improvements in patient-reported outcomes, and SIRIUS established its ability to reduce maintenance OCS while maintaining asthma control (71, 72). In addition, MESILICO supports benefits in late-onset disease with fixed airflow limitation, with improvements in physiological measures and findings consistent with attenuation of airway remodeling (73). For benralizumab, SIROCCO and CALIMA showed significant reductions in exacerbations with improvement in lung function and symptom-related outcomes, and ZONDA demonstrated substantial OCS reduction with sustained clinical benefit (74–76). Depemokimab, an ultra–long-acting anti–IL-5 antibody designed for twice-yearly dosing, has also shown clinically meaningful exacerbation reductions in replicate phase 3 trials (SWIFT-1 and SWIFT-2), supporting the feasibility of long-interval IL-5 blockade as an option to reduce treatment burden while maintaining efficacy (77). In contrast to mepolizumab and benralizumab, for which maintenance OCS-sparing effects have been established in dedicated trials, published evidence specifically demonstrating OCS reduction with depemokimab is not yet available, although its OCS-sparing potential remains of interest for future investigation.

Beyond these standard endpoints, imaging-based assessments suggest that IL-5 pathway inhibition can reduce mucus plug burden and contribute to mucus plug clearance with associated improvements in airflow limitation (78, 79). Real-world cohorts further support sustained reductions in exacerbations and steroid exposure, with continued improvements in asthma control and quality of life over time.

Together, these results support a central pathogenic role for IL-5 and eosinophils in severe eosinophilic asthma. At the same time, multi-omics studies suggest that, when the response to IL-5-targeted therapy is incomplete, factors other than IL-5 may also contribute to persistent eosinophil survival and activation.

### 5.2 CRSwNP

CRSwNP is a chronic inflammatory disease of the sinonasal mucosa characterized by polyp formation, nasal obstruction, and recurrent symptoms, and it is frequently associated with type 2 inflammation and tissue eosinophilia.

In CRSwNP, IL-5 pathway blockade can improve both objective disease burden and key symptoms. In the phase 3 SYNAPSE trial, mepolizumab significantly reduced endoscopic nasal polyp size and improved nasal obstruction compared with placebo on top of standard of care, supporting IL-5 as a clinically relevant driver in a substantial subset of patients (80). Depemokimab also met co-primary endpoints in the replicate phase 3 ANCHOR-1 and ANCHOR-2 trials, demonstrating sustained reductions in nasal polyp score and improvements in nasal obstruction through 52 weeks, consistent with durable IL-5 suppression with a low treatment frequency (81).

Beyond IL-5, strong efficacy signals with other pathways highlight additional, and sometimes dominant, disease biology in CRSwNP: dupilumab improved nasal polyp score and nasal congestion/obstruction (with broad improvements in radiologic and patient-reported outcomes) in the phase 3 SINUS-24 and SINUS-52 trials, underscoring the contribution of IL-4/IL-13 signaling (82, 83). More recently, tezepelumab demonstrated significant improvements in nasal polyp severity and nasal congestion and markedly reduced the need for surgery and systemic corticosteroids in the phase 3 WAYPOINT trial, supporting an important role for upstream epithelial cytokines such as TSLP in driving CRSwNP (84).

Collectively, these results indicate that while IL-5–eosinophil biology is therapeutically actionable in CRSwNP, IL-4/IL-13 and TSLP pathways can also contribute substantially, thereby providing a rationale for mechanism-guided selection and sequencing of biologics.

### 5.3 EGPA and HES

EGPA, characterized by adult-onset asthma with marked eosinophilia and systemic necrotizing vasculitis affecting multiple organs, often with or without MPO-ANCA positivity, is a systemic eosinophilic disease. In EGPA, IL-5 pathway blockade has demonstrated clinically meaningful benefits in pivotal trials. In the phase 3 MIRRA trial, mepolizumab increased accrued remission and reduced relapse burden while enabling glucocorticoid dose reduction, establishing IL-5–targeted therapy as an effective option for relapsing or refractory disease (85). More recently, the phase 3 head-to-head MANDARA trial showed that benralizumab was noninferior to mepolizumab for remission induction, further supporting IL-5–eosinophil biology as a shared therapeutic axis across EGPA phenotypes (86). Depemokimab is being evaluated in EGPA in the phase 3 OCEAN trial (NCT05263934) to test whether twice-yearly dosing is noninferior to mepolizumab in achieving sustained remission and reducing oral corticosteroid use.

In addition to these trial-level outcomes, emerging clinical evidence suggests broader immunologic effects: improvement of peripheral neuropathy has been reported in observational studies and case-based analyses during mepolizumab treatment, consistent with clinically relevant modulation of eosinophil-driven tissue injury programs (87). Furthermore, declines in MPO-ANCA titers have also been reported in selected patients treated with mepolizumab, pointing to potential links between IL-5–conditioned eosinophil activation and autoantibody-associated pathways (88). Taken together, these findings support the view that IL-5 sits within a wider immune network and helps maintain eosinophil activation even when vasculitic or autoantibody-driven processes are also present.

HES comprises a heterogeneous group of disorders defined by persistent hypereosinophilia with eosinophil-mediated organ damage, spanning reactive, idiopathic, and clonal variants. HES is another systemic eosinophilic disorder in which IL-5 pathway targeting yields clear clinical benefit. In a phase 3 randomized, placebo-controlled trial (NCT02836496), mepolizumab significantly reduced disease flares and improved overall disease control with no new safety signals, supporting its role as an effective add-on therapy for patients with recurrent flares (89). Benralizumab has also shown efficacy in PDGFRA-negative HES: in a randomized, double-blind, placebo-controlled phase 2 trial (NCT02130882), benralizumab produced near-complete eosinophil depletion with associated clinical responses in a substantial proportion of patients (90). Phase 3 NATRON (NCT04191304) has also reported delayed time to first HES worsening/flare with benralizumab. The phase 3 DESTINY trial (NCT05334368) is testing whether depemokimab, added to standard of care, reduces HES flare frequency compared with placebo.

### 5.4 Other eosinophilic lung diseases (ABPA/ABPM and CEP)

Among eosinophilic lung diseases, two clinically important entities are ABPA/ABPM and CEP, both of which commonly occur in patients with asthma. ABPA/ABPM is a fungal-associated, type 2-high airway disease characterized by eosinophil-rich mucus plugging and central bronchiectasis (91), whereas CEP is an eosinophilic inflammatory pneumonia involving the lung parenchyma that is typically corticosteroid responsive but relapse prone (92).

At present, anti–IL-5 and anti–IL-5Rα biologics are not approved specifically for ABPA/ABPM or CEP, and large placebo-controlled randomized trials are not available; therefore, the current evidence base relies mainly on observational cohorts and case series.

In ABPA/ABPM, published cohorts and case series have reported that IL-5 pathway blockade (mepolizumab or benralizumab) can reduce mucus plug burden and, in some patients, lead to marked decreases or disappearance of mucus impaction. These reports also describe fewer relapses/exacerbations, improved lung function, and facilitation of systemic corticosteroid tapering in steroid-dependent or frequently relapsing cases (93).

In CEP, small case series suggest that IL-5 pathway biologics may improve eosinophilic pneumonia activity, help prevent relapse, and support corticosteroid tapering in recurrent or steroid-dependent cases (94).

Together, these real-world observations support the concept that IL-5 contributes to eosinophil activation even in disease settings shaped by antifungal immune responses and that IL-5 may promote eosinophilic inflammation not only in the airways but also within the lung parenchymal/interstitial compartment.

## 6 Conclusions and future perspectives

IL-5 contributes to the amplification of type 2 inflammation through its diverse actions on eosinophils. Although SEA, CRSwNP, EGPA, HES, and other eosinophilic diseases share a type 2-inflamed foundation, the target organs and immune environments differ by condition, shaped by factors such as allergen exposure, microbial responses, and coexisting autoimmune inflammation. Within these contexts, IL-5-conditioned eosinophil responses may be further enhanced by pro-inflammatory cues, including IL-4/IL-13, epithelial alarmins such as IL-33, pattern-recognition receptor signaling, and IFN-γ—whereas type I interferons and retinoic acid may counter-regulate these programs. Even in this heterogeneous landscape, targeting IL-5 as a key bottleneck in eosinophil activation is a rational and effective strategy to control disease activity in many patients with these eosinophilic disorders.

Advancing precision medicine toward sustained remission will require improved tools to quantify IL-5-dependent activity using accessible biomarkers such as blood and sputum eosinophil counts, eosinophil granule proteins, and markers of ETosis such as galectin-10. The development and prospective validation of such biomarker and profiling strategies will be essential for optimizing patient stratification, therapy selection, and treatment sequencing toward long-term disease control.
